# Infectious Endocarditis by Pseudomonas aeruginosa in an Immunocompetent Adult

**DOI:** 10.7759/cureus.35072

**Published:** 2023-02-16

**Authors:** Ioanna I Yglesias Dimadi, Marcelo Rodríguez Murillo, Manuel A Villalobos Zúñiga

**Affiliations:** 1 General Practice, Universidad de Costa Rica, San Jose, CRI; 2 Cardiology, Hospital San Juan de Dios, Caja Costarricense del Seguro Social, San Jose, CRI; 3 Infectious Diseases, Hospital San Juan de Dios, Caja Costarricense del Seguro Social, San Jose, CRI

**Keywords:** immunocompetence, bacteremia, nosocomial infection, infectious endocarditis, pseudomonas aeruginosa

## Abstract

In the following case review, we present a 49-year-old male without a history of injection drug (IDU) use nor any known structural heart disease, who developed left-sided pseudomonal infectious endocarditis. The only known risk factors were urinary tract infection (UTI) with secondary bacteremia and prolonged healthcare contact with admission to the intensive care unit.

Infectious endocarditis (IE) is the infection of the endocardium. The official diagnosis can only be established after histological and microbiological studies confirm microorganism-colonized vegetations in the heart valves, but a clinical suspicion with high sensitivity and specificity can be approached with modified Duke’s criteria. Even though structural heart disease is the major predisposing factor for IE, healthcare-associated IE has risen with the new therapeutic interventions. Transient bacteremia, which might result after various procedures, forms part of the factors causing healthcare-associated IE.

Although both, community-acquired and hospital-acquired infections by *Pseudomonas aeruginosa* have been reported, pure community-acquired infections without previous exposure to the hospital or healthcare environment are extremely rare. Intensive care unit (ICU) patients are at special risk for this microbe. It is considered an important causative agent in ventilator/associated pneumonia, catheter-associated urinary tract infection (UTI), and catheter-associated bloodstream infections.

IE by *P. aeruginosa* remains a rare form of IE. Though 95% of cases are associated with injection drug use (IDU), healthcare contact is becoming more important each day as the primary risk factor. The most common complications include abscesses in the ring and annulus, congestive heart failure (CHF), embolisms, inability to sterilize valves, splenic abscesses, recurrent bacteremia, and neurologic complications. This condition is highly fatal, with a mortality rate of over 73% for patients older than 30 years.

Recommended antibiotic treatment for IE caused by *P. aeruginosa* consists of high-dose tobramycin in combination with antipseudomonal penicillin or high-dose ceftazidime, cefepime, or imipenem.

## Introduction

Infectious endocarditis (IE), as the name describes, is the infection of the endocardium. The endocardial surface of the heart valves is the place more commonly affected. Historically, *Staphylococcus aureus* is the leading causative pathogen. This disease occurs more commonly in men with a male-to-female ratio of 1.7 to 1 [1].

Until not long ago, injection drug use (IDU) was the principal risk factor for enteric gram-negative IE, which is the focus of this article. Nevertheless, recent studies have shown that health care contact has become the main risk factor to acquire enteric gram-negative IE. Healthcare-associated IE is especially important for this article as it is the probable source of infection in our case. This form of IE has risen with new therapeutic interventions such as catheters, dialysis shunts, etc [1]. In the prospective cohort study by Benito et al. (2009), healthcare-associated IE was attributed to 34% of 1622 cases of native valve endocarditis and no previous history of IDU [2,3].

We believe the importance of this case report resides in its oddity and diagnostic difficulty. We hope it helps other physicians have this diagnosis in mind when it comes to nonspecific clinical characteristics such as the ones that our patient presented.

## Case presentation

The patient of this case is a 49-year-old male with recurrent urinary tract infections (UTIs); managed in the primary care (of the Caja Costarricense del Seguro Social or CCSS) for hypertension, carbohydrate intolerance, and obesity; known as a former smoker and alcohol consumer; with an allergy to sulfa-drugs and with no other relevant medical hereditary background. He had no history of previous drug use of any kind nor sexually transmitted diseases (STDs).

The patient was first consulted by the emergency department (ED) of a national public hospital (of the CCSS) for acute onset of upper urinary tract symptoms. At this time, the patient had no symptoms nor signs of systemic compromise. An obstructive lithiasis of 40x16x16 mm was documented in the left ureteropelvic junction and urine culture was positive for multidrug-sensitive *Pseudomonas aeruginosa*. The abdominal ultrasound showed left hydronephrosis. Antibiotic coverage was empirically started with Cefotaxime. Due to his obstructive uropathy, the patient underwent Double-J catheter placement.

In the days after the procedure and during the recovery, the patient became tachycardic with hypotension, requiring vasopressors. The diagnosis of septic shock was made. Blood cultures resulted positive for multidrug-sensitive *P. aeruginosa*, and antibiotic coverage was changed to Amikacin and Ceftazidime. After 13 days the patient continued to evolve unsatisfactorily, maintaining the need for vasopressors. The antibiotic treatment was changed to Meropenem. Imaging studies showed Double-J catheter misplacement, so the patient was taken again to the de operating room (OR) for an open pyelolithotomy through a lumbotomy with Double-J stent replacement.

In the days following the Double-J catheter replacement, the patient developed respiratory failure and hypotension. Studies documented a new complicated right basal pneumonia with pleural effusion. Antibiotic coverage was again titrated, this time to Piperacillin-tazobactam based on antibiogram results. Because of a lack of response, the patient was placed on mechanical ventilation and taken to the intensive care unit (ICU). Antibiotic coverage was empirically amplified to Meropenem, Levofloxacin, and Vancomycin. The patient was finally extubated after 11 days on MV. New blood cultures were negative. After recovery, the patient was discharged.

A week later the patient was brought back to the ED with shortness of breath and a fever over 40°C. On physical examination, the patient presented prolonged capillary refill time, tachycardia, dyspnea, dry mucous membranes, and no heart murmurs. A chest x-ray showed a new consolidation. A computed tomography (CT) of the chest confirmed these findings and the diagnosis of atypical pneumonia was made.

**Figure 1:**
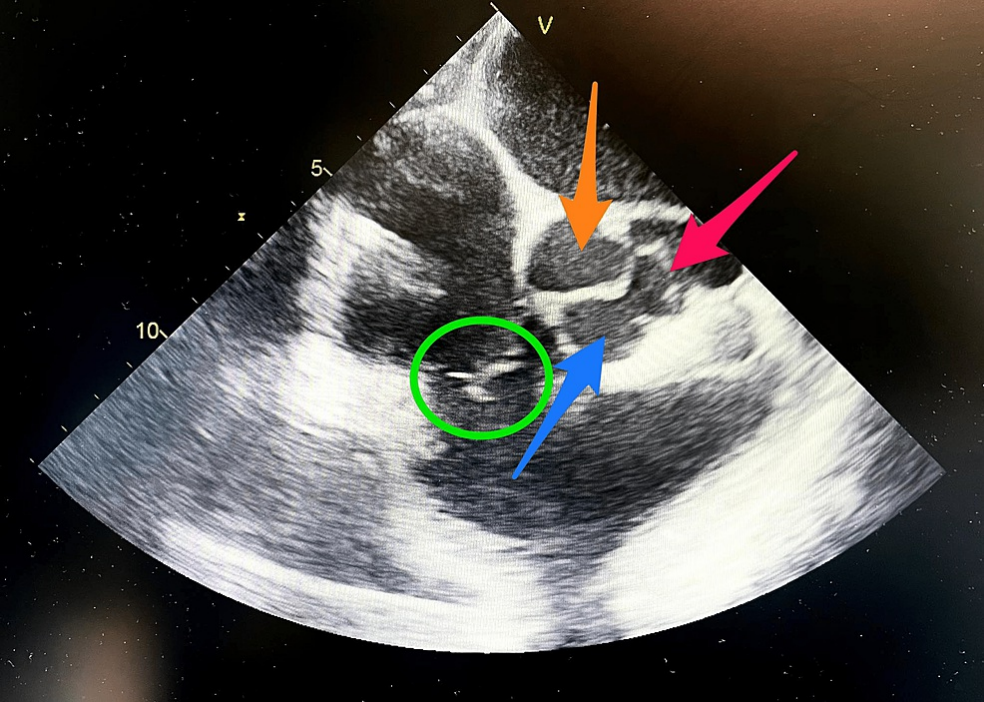
Transesophageal echocardiogram at 60 degrees. Short axis view of aortic valve shows perforation and rupture of left coronary leaflet. Green circle shows the tricuspid valve. Blue arrow shows the right coronary cusp of aortic valve. Pink arrow shows left coronary cusp of aortic valve with teared left coronary leaflet. Orange arrow shows non-coronary cusp of aortic valve.

**Figure 2:**
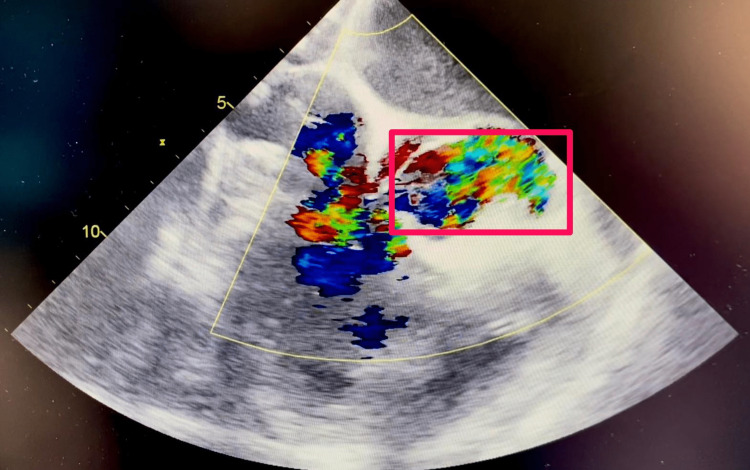
Color doppler transesophageal echocardiogram. Pink square shows an eccentric jet secondary to severe aortic insufficiency through the left coronary leaflet.

Days later, the patient evolved to septic shock with respiratory failure and required mechanical ventilation with constant vasopressors. Several new blood cultures were positive for *P. aeruginosa*. On physical examination were documented a new holosystolic aortic heart murmur that radiated to carotid arteries, a diastolic murmur in the mitral auscultation point, and clinical signs of heart failure. The transesophageal echocardiogram (TEE) showed severe aortic valve regurgitation with an aortic valve root abscess, along with vegetations in the mitral-aortic complex root, and compromise of the anterior mitral valve leaflet (Figures 1, 2). The patient was programmed for surgical intervention.

**Figure 3:**
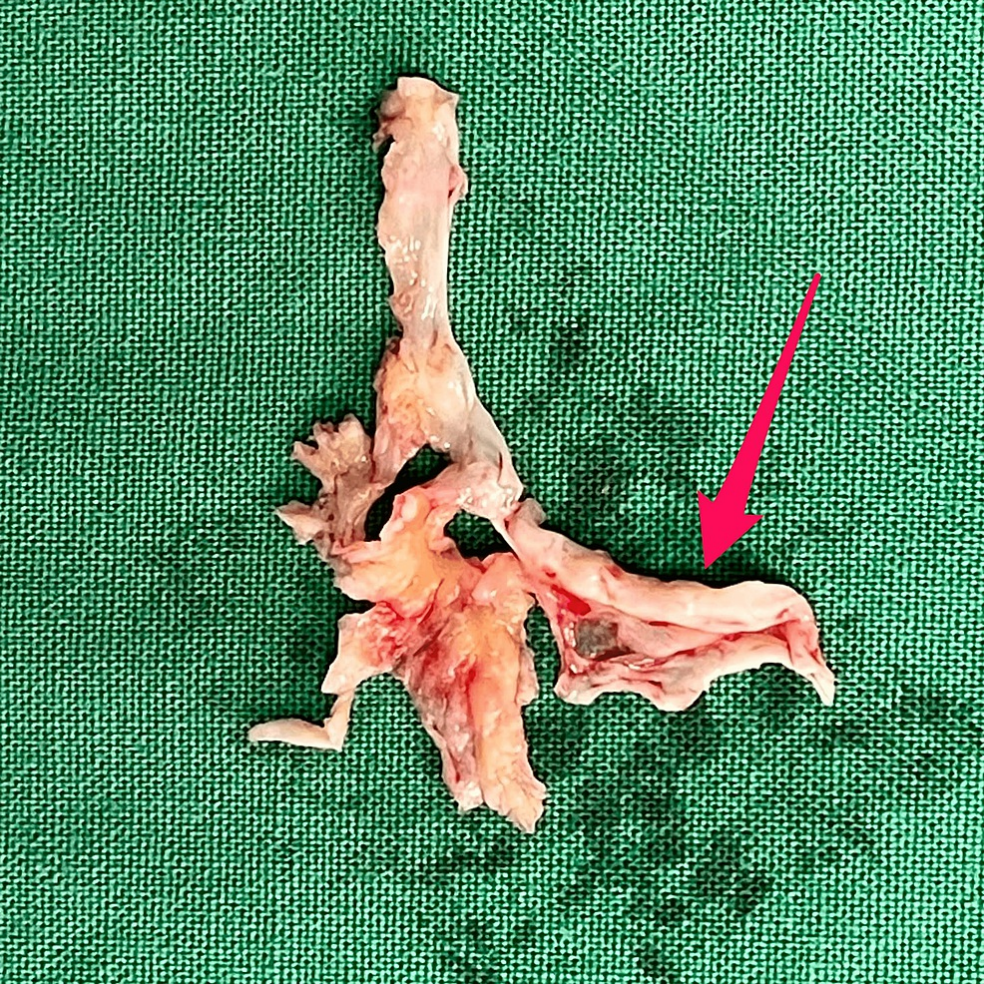
Pathology specimen of the aortic valve after surgical resection. Pink arrow shows complete destruction of the left coronary leaflet along with vegetations.

The presurgical diagnosis was subacute bacterial endocarditis with severe aortic regurgitation and aortic root abscess. Surgical findings were a normal aorta and aortic root, complete rupture of the left coronary aortic sinus, multiple vegetations in the left coronary aortic sinus and the non-coronary sinus, and partial detachment of the anterior mitral sinus (Figure 3). The aortic valve was replaced with a mechanic supra-annular valve and the mitral leaflet was reattached.

Unfortunately, during surgery, the patient suffered from a septic embolus to the anterior descending artery territory and went into cardiogenic shock. The patient had a poor evolution after surgery requiring inotropes at high doses and intra-aortic balloon counterpulsation. Despite all measures taken, the patient remained in cardiogenic shock and died one day after surgery.

## Discussion

Literature review

During the literature review, previous case reports of *P. aeruginosa* left-sided IE not related to IDU were searched. Although the number of reported cases with these characteristics was very limited, many shared clinical aspects with our case.

General aspects of infectious endocarditis

When the suspicion of IE is made in a febrile patient presenting with a new or different heart murmur: clinical, microbiologic, and echocardiographic evaluations should be performed. The official diagnosis can only be established after histological and microbiological studies confirm microorganism-colonized vegetations in the heart valves, but a clinical suspicion with high sensitivity and specificity can be approached with modified Duke’s criteria [4]. In our case the definitive clinical diagnosis was established after documenting both major Duke’s criteria: two blood cultures drawn more than 12 hours apart resulted positive for *P. aeruginosa*; plus evidence in TEE and/or transthoracic echocardiogram (TTE) of new endocardial involvement.

Besides the important role that plays extensive contact with the health care system in developing IE, structural heart disease of any type remains the most important predisposing factor [1]. In our case, there is no previous history of structural heart disease. The patient’s heart murmur was documented after blood cultures resulted positive.

Transient bacteremia, which might result after various procedures, forms part of the factors causing healthcare-associated IE. It occurs when a mucosal surface colonized by bacteria is traumatized during a procedure or after its manipulation [5]. The more trauma produced and the number of microorganisms colonizing the mucosa, the more the degree of bacteremia. Organisms causing transient bacteremia usually correspond to the normal resident flora in the traumatized mucosa. Some examples of the percentage of positive blood cultures after certain procedures are as follows: 16% after intubation, 18% to 33% after urethral dilation, and 8% after urethral catheterization. It is important to clarify that only bacteria resistant to the bactericidal activity of the complement-mediated immune response, like *P. aeruginosa*, can evolve into IE after transient bacteremia [1].

In one of the reviewed cases, similar to ours, the patient had a history of several cystoscopic procedures for obstructive uropathy, summed to numerous episodes of UTIs by *P. aeruginosa* in the past months, which led to bacteremia and IE by this pathogen. According to Dawson et al., genitourinary sources are the second most frequent factor for IE in patients without IDU history as it is a common pathogen in the urinary tract and invasive urologic procedures could cause its translocation [6]. This takes importance in our case, because of our patient’s previous genitourinary problems.

Gram-negative bacilli

IE by this group of bacteria causes persistent bacteremia despite high levels of antimicrobials and congestive heart failure (CHF) commonly occurs. The prognosis is usually poor [1]. The physician should suspect IE when a patient develops a new heart murmur during gram-negative sepsis, summed to unexplained anemia and persistent positive blood cultures.

Overview of *P. aeruginosa*

*P. aeruginosa* is a ubiquitous obligate aerobic gram-negative rod. It forms colonies with a grapelike odor and green-blue color. It is classified as oxidase positive. Several virulence factors have been found in these bacteria that play an important role in their pathogenesis. Some of these factors implied are pili and flagella for attachment, motility, and biofilm formation; secretion of toxins in the extracellular space; a secretion system for cytotoxicity and colonization; endotoxins to resist the innate immune response; among other factors [7]. *P. aeruginosa* is difficult to eliminate and can survive on objects and surfaces due, in part, to its ability to form biofilm.

The relationship that a microbe establishes with the Human body can be classified according to the type of this relationship into commensal, pathogen, obligate pathogen, commensal pathogen, zoonotic pathogen, and environmental pathogen. *P. aeruginosa* is considered an environmental pathogen as it is acquired by Humans from water or soil and causes disease accidentally, but this process is not necessary for the survival of the microbe. Moreover, *P. aeruginosa *represents an intermediate between a primary and opportunistic pathogen as it can cause disease in both immunocompetent and immunocompromised patients, varying the severity of infection, site of affection, and mechanism of dissemination [8].

Epidemiology of P. aeruginosa

Although both, community-acquired and hospital-acquired infections by *P. aeruginosa* have been reported, pure community-acquired infections without previous exposure to the hospital or healthcare environment are extremely rare. ICU patients are at special risk for this microbe [7]. It is now considered the most common pathogen causing ventilator-associated pneumonia [9], the third most common cause of catheter-associated UTI, and the 10th cause of catheter-associated bloodstream infections [7].

Acquisition from the hospital environment usually occurs through contaminated healthcare personnel hands and hospital surfaces, and the most common reservoirs usually involve humidity or water. Approximately 20.5% of healthcare worker-patient interactions result in contamination of the healthcare worker’s gloves or gowns. *P. aeruginosa* is the second most common bacteria causing this type of contamination. Risk factors for healthcare worker contamination include positive environmental cultures, duration in the room for more than 5 minutes, performing a physical exam, and contact with the ventilator [10].

Infections caused by P. aeruginosa

Urinary tract infections: *P. aeruginosa* is one of the most common pathogens associated with nosocomial UTIs. This type of infection usually implies an underlying comorbidity like diabetes mellitus. As it normally occurs in patients with medical history of prostatitis, lithiasis, neurogenic bladder and urologic instrumentations; this condition is usually related with health care contact. Other risk factors include male gender, antimicrobial use and ICU admission [11,12].

Bacteremia: according to Wisplinghoff et al., as cited in Dolind, *P. aeruginosa* is the third most common gram-negative bacteria causing nosocomial bacteremia and its mortality rate is one of the highest [13]. Risk factors mainly include previous hospitalization or antimicrobial drug use, surgery, invasive devices, advanced age and immunodeficiency [14,15].

Our patient’s medical history is crucial when solving this case. He had a clear previous history of UTIs and obstructive uropathy, summed to multiple healthcare contacts in the months previous to his hospitalization for the same cause. Also, although he only had history of carbohydrate intolerance, his blood glucose levels during his hospital stay oriented more towards an undiagnosed type 2 diabetes mellitus. The patient developed pseudomonal bacteremia after being admitted to the hospital and undergoing an urologic procedure in a mucosa colonized by *P. aeruginosa*. Also, it was until after the first blood cultures resulted positive for* P. aeruginosa*, that a new heart murmur was noted on physical examination.

Infectious endocarditis by *P. aeruginosa*

IE by *P. aeruginosa* remains a rare form of IE, accounting for only 3% of all cases. It is considered part of the non-HACEK pathogens to cause endocarditis. Epidemiology, then, plays an important role helping the physician make the clinical suspicion. Though 95% of cases are associated with IDU, health care contact is becoming more important each day as the primary risk factor. The mean age of disease is 30 years and it is 2.5 times more common in males than in females [1,3].

The most common complications include abscesses in the ring and annulus, CHF, embolisms, inability to sterilize valves, splenic abscesses, recurrent bacteremia, and neurologic complications. It is important to note that classic skin manifestations of endocarditis, like Osler nodes and Janeway lesions, are usually absent in pseudomonal IE [6].

This condition is highly fatal, with a mortality rate over 73% for patients older than 30 years. Left-sided involvement has the worst prognosis [1]. Left sided IE by *P. aeruginosa* in non-drug users primarily occurs as a nosocomial infection after an invasive procedure. It can also occur in patients with infected intravascular devices. It is important to state that underlying heart valve disease is not necessary for this condition to occur [6].

Our patient then, although rare, matches the epidemiologic profile. As he had no history of IDU, his prolonged hospital stay, along with the multiple invasive procedures he underwent, play an important role in his disease. Finally, our patient’s major complications were CHF, requiring constant norepinephrine; and left-side involvement, making his management especially difficult.

Treatment

Recommended antibiotic treatment for IE caused by *P. aeruginosa *consists of high-dose tobramycin (8 mg/kg/day IV or in divided doses, given intramuscular) in combination with an antipseudomonal penicillin (like piperacillin, ticarcillin, azlocillin) or high-dose ceftazidime, cefepime or imipenem. Treatment must be given for six to eight weeks [1].

Antibiotic treatment alone has proven to be ineffective in most left-sided pseudomonal IE. Studies have shown that the possible explanation for this is that antibiotic concentrations, when using ceftazidime and tobramycin, are more often below the minimum bactericidal concentration in aortic valve vegetations compared to tricuspid valve vegetations. Also, it has been suggested that treatment failure in absence of surgery might be due to the high density of microorganisms in the vegetations [6]. Due to the high rate of failure with medical therapy alone in left-sided IE caused by this pathogen, prompt surgical intervention is necessary to attempt for survival [6].

## Conclusions

Left-sided infectious endocarditis by *P. aeruginosa* in patients without a history of IDU is rare; especially in patients without underlying structural valvular anomalies. Clinical suspicion should rise in patients with a history of genitourinary pathology, invasive procedures, and close healthcare contact. Prompt antibiotic treatment along with surgical intervention is necessary to attempt survival.
